# Effect of silymarin on blood coagulation profile and osmotic fragility in carbon tetrachloride induced hepatotoxicity in male Wistar rats

**DOI:** 10.1016/j.toxrep.2022.06.005

**Published:** 2022-06-09

**Authors:** Aminat Bolanle Popoola, Emmanuel Oluwaseun Ademilusi, Temitope Gabriel Adedeji, Adesoji Adedipe Fasanmade

**Affiliations:** a Department of Physiology, Faculty of Basic Medical Sciences, University of Ibadan, Ibadan, Nigeria; b Department of Physiology, Federal University of Technology Akure, Akure, Nigeria; c Medicine Department, University College Hospital, Ibadan, Nigeria

**Keywords:** AI, atherogenic Index, ALP, alkaline phosphatase, ALT, alanine aminotransferase, aPTT, activated partial thromboplastin time, AST, aspartate aminotransferase, CAT, catalase, CCl_4_, Carbon tetrachloride, CYP, cytochrome P 450, DNA, deoxyribonucleic acid, GSH, glutathione, HDL, high density lipoprotein cholesterol, INR, international normalized ratio, ISI, international sensitivity index, LDL, low density lipoprotein cholesterol, MDA, malondialdehyde, PT, prothrombin time, RLW, relative liver weight, S, silymarin, SEM, standard error of mean, SOD, superoxide dismutase, TC, total cholesterol, TT, Thrombin time, liver, oxidative stress, dyslipidemia

## Abstract

Reports about the impact of Carbon tetrachloride (CCl_4_) hepatotoxicity on coagulation profile have been inconsistent. Multiple investigators have however demonstrated the effectiveness of silymarin in the resolution of anomalies induced by CCl_4_, although the effect of silymarin on the impact of CCl_4_ hepatotoxicity, especially coagulation profile and osmotic fragility have not been investigated. The liver, the primary site for the secretion of coagulation proteins, can become impaired in CCl_4_ hepatotoxicity, and silymarin reportedly increases hepatic protein synthesis as part of its hepatoprotective mechanism. This study assessed the effect of silymarin on blood coagulation profile and erythrocyte osmotic fragility in CCl_4_ induced hepatotoxicity in rats. Twenty male Wistar rats were allocated into four groups (n = 5) at random, namely: Control, CCl_4_ given CCl_4_ (1 ml/kg) administered intraperitoneally twice a week, Silymarin (S) given silymarin (100 mg/kg/day) orally, and S+CCl_4_ given silymarin (100 mg/kg/day) orally and (1 ml/kg) CCl_4_ one hour after, intraperitoneally twice a week for a duration of four weeks. Results showed protraction of activated partial thromboplastin time and thrombin time, increased erythrocyte osmotic fragility, liver damage, dyslipidemia, oxidative stress and lipid peroxidation in rats given CCl_4_. Silymarin attenuated most of these effects as observed from comparison between CCl_4_ and S+CCl_4_ rats. The findings of this study suggests that pretreatment with silymarin attenuated disruption in coagulation profile and erythrocyte osmotic fragility in CCl_4_ induced hepatotoxicity in Wistar rats.

## Highlights

• Carbon tetrachloride induced hepatotoxicity disrupts intrinsic pathway of blood coagulation.
• Sub-acute carbon tetrachloride administration causes erythrocyte osmotic fragility.
• Silymarin attenuates toxic effect of carbon tetrachloride on blood coagulation.

## Graphical Abstract

**Figure:**
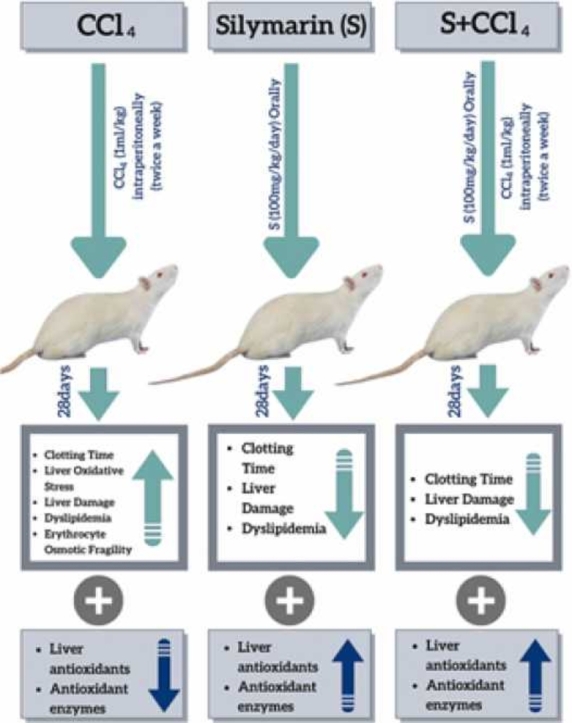

## 1 Introduction

Carbon tetrachloride (CCl_4_) is a colorless, non-flammable environmental pollutant that causes tissue necrosis and cell damage [1]. Carbon tetrachloride is employed for induction of liver damage in experimental models [2]. It has been used as a model hepatotoxicant for toxicology studies in both in-vitro and in-vivo animal models, and to study hepatoprotective activity of drugs and herbs against liver damage. Carbon tetrachloride undergoes phase 1 metabolism in the endoplasmic reticulum of the liver by cytochrome P 450 (CYP) enzymes (CYP 2E1, CYP2E and possibly CYP3A) to form trichloromethyl radical or forms trichloromethylperoxyl radical with oxygen, which is a very strong reactive oxygen specie. These radicals cause liver injury by binding to cellular molecules and thus damaging vital cellular processes. These includes peroxidation of lipid in the membrane bilayer and oxidation of membrane proteins and other cell components such as deoxyribonucleic acid (DNA) and Ribonucleic acid [3]. It also leads to hepatic fatty degeneration and inflammation in the acute stage of liver injury. Fibrosis and cirrhosis of the liver occurs in the chronic stage of liver injury.

The liver is the main site for the production of blood coagulation factors like factors II (prothrombin), I (fibrinogen),VI, VII, IX, XI, proteins C and S, and antithrombin [4]. Impaired blood coagulation due to liver dysfunction has different etiologies ranging from impaired coagulation factor synthesis, increased clearance of coagulation factors from the circulation and others. Coagulation disorders owing to hepatic disease are usually measured by elongation of prothrombin time (PT) and activated partial thromboplastin time (aPTT) [5].

Silymarin is gotten from milk thistle *(Silybum marianum)* seed. It is well known as a standard hepatoprotective drug in the treatment of CCl_4_ induced hepatotoxicity. It has also been used in comparison with various hepatoprotective agents in assessing the effectiveness of these agents. Different studies have reported the effectiveness of silymarin in the resolution of oxidative stress, fibrosis, elevated liver enzymes, lipid peroxidation and necrosis caused by CCl_4_, but the effect of silymarin on CCl_4_ induced hepatotoxicity on coagulation profile and osmotic fragility has not been investigated. The liver, being the primary site for secretion of coagulation proteins, can become impaired in diseased states, and silymarin has been reported to increase hepatic protein synthesis as part of its hepatoprotective mechanisms [6]. This study therefore investigated silymarin’s effect on blood coagulation profile and erythrocyte osmotic fragility in CCl_4_ provoked hepatotoxicity in male Wistar rats.

## 2 Materials and methods

### 2.1 Chemicals and reagents

Carbon tetrachloride BDH Analar® Prod.100 745 R, sodium citrate, olive oil, silymarin tablets (silybon-140) from Micro Labs Limited (India). Assay kits used for superoxide dismutase (SOD) and lipid profile (lipoproteins, total cholesterol, triglyceride) were gotten from Fortress Diagnostic (Antrim, UK) while those used for alanine aminotransferase (ALT), alkaline phosphatase (ALP), and aspartate aminotransferase (AST) were obtained from Elabscience Biotechnology Inc, USA. Malondialdehyde (MDA) assay kit was obtained from Oxford Biomedical Research, Inc. (USA) while those used for the determination of glutathione (GSH), catalase (CAT), and nitrite were gotten from Elabscience Biotechnology Company, Ltd., Wuhan, China.

### 2.2 Animals

Twenty male Wistar rats with weight between 150 and 200 g were procured from a local breeder at the University of Ibadan, Nigeria. The animals were acclimatized for two weeks at the Department of Physiology’s Postgraduate Research Animal House, University of Ibadan. They were kept in polythene cages throughout the period of the study and allowed access to feed and water ad-libitum. Humidity and room temperature were kept constant within standard limits and 12-hour light/dark cycle was maintained. The experimental procedures were endorsed by University of Ibadan Animal Care and Use Research Ethics Committee (UI-ACUREC/20/008). The procedures of the experiment also conform to the National Institutes of Health Guide for the Care and Use of Laboratory Animals National Research Council US [7], and the study is reported in conformity with Animal Research: Reporting of *In Vivo* Experiments guidelines [8].

### 2.3 Hepatotoxicity induction and silymarin administration

Liver toxicity was induced using Carbon tetrachloride (1 ml/kg body weight). Carbon tetrachloride was dissolved in equal volume of olive oil (1:1 v/v ratio) and given intraperitoneally two times a week for 4 weeks [9]. Silymarin (100 mg/kg) was administered orally once daily via oral gavage [10]. Silymarin was dissolved in distilled water according to w/v ratio.

### 2.4 Experimental design

The rats were all fed the standard rat chow (Ladokun Feed) and were allocated into 4 groups (n = 5) at random, namely: Control (group 1), CCl_4_ (group 2), Silymarin (S) (group 3) and S+CCl_4_ (group 4). Group 1 was given distilled water, group 2 was given CCl_4_ (1 ml/kg) administered intraperitoneally twice a week, group 3 was given silymarin (100 mg/kg/day) orally and group 4 was given silymarin (100 mg/kg/day) orally followed by CCl_4_ (1 ml/kg) one hour after, intraperitoneally, twice a week.

### 2.5 Determination of body weight

The body weight of the animals were measured at baseline and weekly using SF-400 electronic weighing scale (Zhejiang Mengxuan Industry & Trade Co., Limited., Jinhua, Zhejiang, China).

### 2.6 Sample collection

At the end of 4 weeks, animals were sacrificed by cervical dislocation. Blood was taken in EDTA coated bottles via the retro-orbital sinus and the liver was harvested for determination of oxidative stress markers and histopathological examination.

### 2.7 Determination of relative liver weight

The liver weight was measured using a sensitive organ weighing scale.

The relative liver weight (RLW) was determined as shown below:

$$\mathrm{Relative}\,\;liver\;weight\;(\%\;\mathrm{body}\;weight)=\;\frac{Liver\;weight}{Body\;weight}\times 100$$

### 2.8 Biochemical assays

Blood cells were separated from plasma as described by Ojetola et al. [11]. High density lipoprotein cholesterol (HDL), total cholesterol (TC), triglycerides, ALT, ALP and AST were determined using commercially available kits in conformity with manufacturer's instructions. However, low density lipoprotein cholesterol (LDL) was determined using the Friedewald equation [12]. Atherogenic index (AI) was estimated as log (triglyceride/HDL) [11].

### 2.9 Determination of erythrocyte osmotic fragility and blood coagulation parameters

Red blood cell osmotic fragility test was done as described by Oyewale [13]. Blood coagulation parameters were determined using standard laboratory methods. International normalized ratio (INR) was determined as follows:

$$\mathrm{INR}={\frac{\mathrm{Test}\;\mathrm{control}}{\mathrm{Test}\;\mathrm{Plasma}}}^{\mathrm{ISI}}$$

ISI is international sensitivity index.

### 2.10 Determination of liver oxidative stress markers

A lobe of liver was cut into pieces and a uniform portion (100 mg) was homogenized in ice cold sodium phosphate buffer (0.1 M, pH 7.4), centrifuged at a temperature of 4 °C for 10 min at 10 000 rpm, and the supernatant was separated. Malondialdehyde was measured as described by Okhawa et al. [14]. Glutathione concentration, nitrite, catalase and superoxide dismutase activity were determined by methods of Jollow et al. [15], Green et al. [16], Goth [17], and Misra and Fridovich [18] respectively.

### 2.11 Hepatic histological examination

**Fig. 1:**
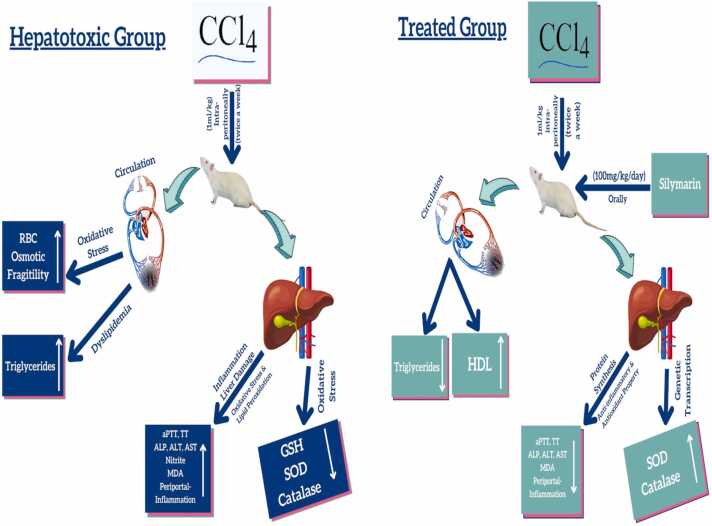
Schematic diagram of in vivo protocol.

This was done as described by [37], [38]. The extent of liver destruction was checked by eosin/hematoxylin staining. The in vivo protocol is shown in Fig. 1.

### 2.12 Statistical analysis

Data were stated as Mean ± Standard Error of Mean (SEM). All data were analyzed with GraphPad prism 7.0 (GraphPad Software, San Diego, CA). One-way analysis of variance was employed for comparison followed by *Post hoc Tukey* multiple comparison test. For variables with initial and final values, two-way analysis of variance was substituted for one-way analysis of variance. *P*<*0.05* was considered statistically significant.

## 3 Results

### 3.1 Silymarin attenuates CCl_4_-induced alterations in coagulation profile

There were significant increases in thrombin time (TT) and aPTT (p = 0.0015 and p = 0.0007 respectively) in CCl_4_ group in comparison with control group, while TT decreased significantly (p = 0.0021) in S+CCl_4_ group in comparison with CCl_4_ group. However, no significant difference was seen in INR and PT across groups when compared with control group (Table 1).

**Table 1:** Effect of silymarin on blood coagulation profile and liver enzymes.

	**Control**	**CCl**_**4**_	**Silymarin**	**S+CCl**_**4**_
**PT (seconds)**	23.0 ± 0.58	22.67 ± 2.19	20.67 ± 0.67	23.67 ± 1.86
**TT (seconds)**	33 ± 4.16	57.67 ± 3.67^a^	32.67 ± 1.67^b^	34.33 ± 0.67^b^
**aPTT (seconds)**	30.33 ± 5.17	150.0 ± 0.0^a^	67 ± 4.0^b^	101.33 ± 24.33^a^
**INR**	1.51 ± 0.04	1.49 ± 0.14	1.49 ± 0.14	1.56 ± 0.12
**ALP (ul**^**-**^**)**	104 ± 8.50	128.67 ± 4.10^a^	103.67 ± 4.10^b^	114 ± 3.06
**ALT (ul**^**-**^**)**	32.0 ± 1.0	43.0 ± 1.53^a^	32.0 ± 0.58^b^	37.33 ± 1.45^b^
**AST (ul**^**-**^**)**	43.33 ± 1.20	54.67 ± 1.76^a^	44.0 ± 1.0^b^	47.67 ± 1.20^b^
**AST/ALT ratio**	1.35 ± 0.01	1.39 ± 0.04	1.37 ± 0.02	1.33 ± 0.05
**RLW (% body weight)**	2.90 ± 0.13	3.07 ± 0.12	2.86 ± 0.05	3.07 ± 0.15

Values were presented as mean ± SEM and n = 5 in each group. Data were analysed using ordinary one-way analysis of variance and post hoc *Tukey test*. ^a^p < 0.05 is significant in comparison with control and ^b^p < 0.05 is significant in comparison with CCl_4_. CCl_4_ = carbon tetrachloride, PT = Prothrombin Time*,* TT = Thrombin Time, aPTT= Activated Partial Thromboplastin Time, INR = International Normalised Ratio, ALP = alkaline phosphatase, ALT = alanine aminotransferase, AST = aspartate aminotransferase, RLW = relative liver weight, S = silymarin.

### 3.2 Silymarin attenuates CCl_4_-induced alteration in liver enzymes

Alkaline phosphatase, ALT and AST increased significantly (p = 0.0468, p = 0.0009 and p = 0.0014 respectively) in CCl_4_ group in comparison with control group while ALT and AST decreased significantly (p = 0.0416 and p = 0.0236 respectively) in S+CCl_4_ group in comparison with CCl_4_ group. However, no significant difference was seen in AST/ALT ratio and RLW across groups in comparison with control (Table 1).

### 3.3 Silymarin attenuates CCl_4_-induced increase in lipid levels

There was significant increase in triglycerides (p = 0.001) in CCl_4_ group in comparison with control group whereas this decreased significantly in S+CCl_4_ group (p = 0.0002) in comparison with CCl_4_ group. HDL also increased significantly (p = 0.0436 and p = 0.0314 respectively) in S+CCl_4_ and Silymarin groups in comparison with CCl_4_ group. However, no significant difference was seen in AI, TC and LDL across groups when compared with control group (Table 2).

**Table 2:** Effect of Silymarin on lipid profile and oxidative stress markers.

	**Control**	**CCl**_**4**_	**Silymarin**	**S+CCl**_**4**_
**Triglycerides (mg/dl)**	46.33 ± 1.33	56.0 ± 0.0^a^	49.33 ± 0.33^b^	43.67 ± 1.67^bc^
**TC (mg/dl)**	78.67 ± 1.33	81.33 ± 0.67	80.0 ± 0.0	81.0 ± 2.00
**LDL (mg/dl)**	26.40 ± 0.6	27.48 ± 1.45	22.47 ± 0.27	24.93 ± 2.40
**HDL (mg/dl)**	43.0 ± 1.0	42.67 ± 1.67	47.67 ± 0.33^ab^	47.33 ± 0.33^b^
**AI**	0.08 ± 0.00	0.07 ± 0.01	0.04 ± 0.02	0.05 ± 0.03
**SOD (U/mg protein)**	119.11 ± 4.26	86.53 ± 2.88^a^	109.75 ± 2.53^b^	104.17 ± 2.88^ab^
**MDA (ηM/mg protein)**	1.44 ± 0.18	4.88 ± 0.79^a^	1.69 ± 0.41^b^	3.38 ± 0.12
**Nitrite (µM/mg protein)**	1.07 ± 0.10	3.38 ± 0.08^a^	1.07 ± 0.10^b^	2.94 ± 0.38^ac^
**Catalase (µ/mg protein)**	58.84 ± 5.46	40.97 ± 0.74^a^	64.39 ± 2.94^b^	56.91 ± 1.67^b^
**Glutathione (µM/mg protein)**	7.90 ± 0.22	5.73 ± 0.21^a^	7.96 ± 0.18^b^	6.43 ± 0.30^ac^

Values were presented as mean ± SEM and n = 5 in each group. Data were analysed using ordinary one-way analysis of variance and post hoc *Tukey test*. ^a^P < 0.05 is significant in comparison with control, ^b^P < 0.05 is significant in comparison with CCl_4_ and ^c^P < 0.05 is significant in comparison with silymarin. CCl_4_ = carbon tetrachloride, TC = total cholesterol, LDL = low density lipoprotein cholesterol, HDL = high density lipoprotein cholesterol, AI = atherogenic index, SOD = superoxide dismutase, MDA = malondialdehyde, S = silymarin.

### 3.4 Silymarin attenuates CCl_4_-induced alterations in oxidative stress markers

There was significant reduction in SOD, catalase and glutathione (p = 0.0004, p = 0.0188 and p = 0.0008 respectively) in CCl_4_ group in comparison with control group, while SOD and catalase increased significantly (p = 0.0193 and p = 0.0336 respectively) in S+CCl_4_ group in comparison with CCl_4_ group. Also, MDA and nitrites increased significantly (p = 0.0033 and p = 0.0002 respectively) in CCl_4_ group when compared individually with control group. However, the decrease in both variables in S+CCl_4_ group was not significant when compared with CCl_4_ group (Table 2).

### 3.5 Changes in body weight

**Fig. 2:**
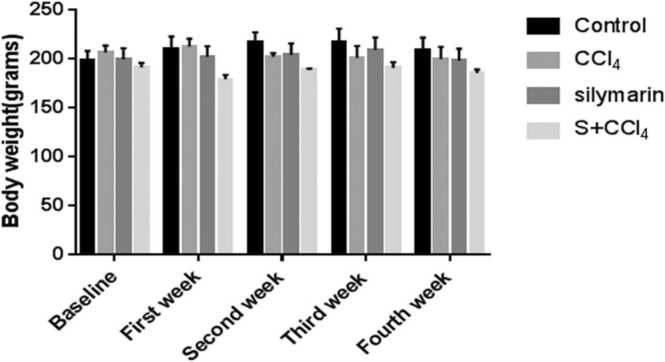
Body weight. Values were presented as mean±SEM and (n = 5). CCl_4_ = carbon tetrachloride and S = silymarin.

No significant difference was seen in body weight across the experimental groups in comparison with control as shown in Fig. 2.

### 3.6 Changes in erythrocyte osmotic fragility

**Fig. 3:**
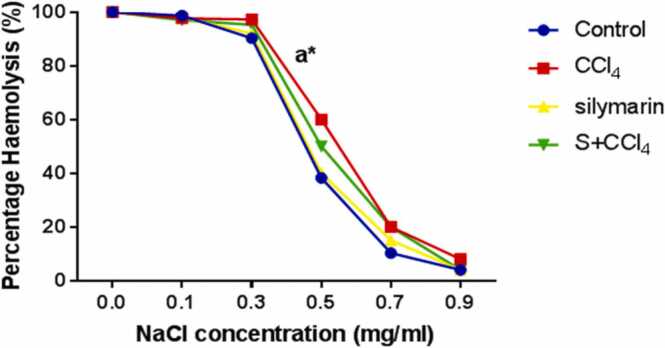
Osmotic fragility curve. Values were presented as mean±SEM and (n = 5). ^a^P < 0.05 is significant in comparison with control. CCl_4_ = carbon tetrachloride, NaCl = Sodium Chloride, S = silymarin.

There was significant increase in percentage haemolysis in CCl_4_ group in comparison with control group at 50% haemolysis. The NaCl concentration at 50% haemolysis in control was 0.453 while 50% haemolysis in CCl_4_ group was 0.551 as shown in Fig. 3.

### 3.7 Histopathological changes in the Liver

**Fig. 4:**
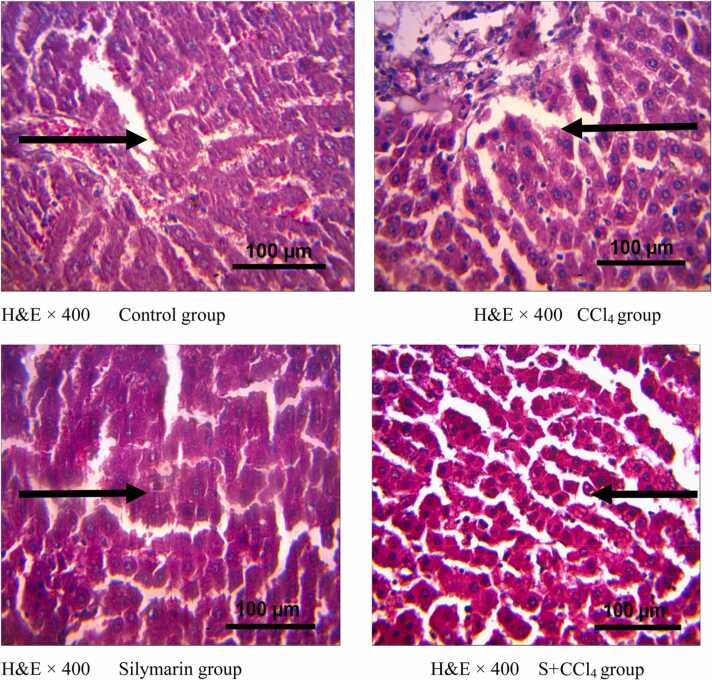
Photomicrographs of the Liver.

The control group showed no visible lesion, but CCl_4_ group showed periportal cellular inflammation with infiltration of cells. The silymarin group showed no visible lesions and S+CCl_4_ group showed no visible lesions as shown in Fig. 4.

## 4 Discussion

The current study was done to assess silymarin’s effect on blood coagulation profile (Prothrombin time, activated partial thromboplastin time, thrombin time, and international normalised ratio) and erythrocyte osmotic fragility in carbon tetrachloride induced hepatotoxic Wistar rats. Subacute administration of CCl_4_ was done for four weeks intraperitoneally twice a week to assess its hepatoxicity on blood coagulation profile and erythrocyte osmotic fragility. Silymarin was also administered daily for twenty eight days alongside CCl_4_ administration to assess its hepatoprotective effect on blood coagulation profile and erythrocyte osmotic fragility in CCl_4_ provoked hepatotoxicity in rats.

The coagulation profile indicates disruption of the intrinsic pathway as evidenced by aPTT and TT prolongation in CCl_4_ group in comparison with control while the results suggested that the extrinsic pathway was not significantly altered as shown by PT and INR values in comparison with control values. This can be adduced to reduction in hepatic production of some factors of the intrinsic pathway as a result of liver damage. Silymarin’s reduction of TT and aPTT is attributable to its ability to increase hepatic protein synthesis through DNA polymerase, which increases DNA synthesis and formation of ribosomes that produce proteins [19], [6] thereby increasing production of clotting factors of the intrinsic pathway.

The significant increase in liver enzymes ALT, AST, and ALP in CCl_4_ group can be directly related to hepatic inflammation as confirmed by liver histology and damage of liver cells, allowing the leakage of intracellular enzymes from cytosol into the blood [20]. Silymarin’s reduction of liver enzymes concentration in circulation is attributed to its ability to reduce inflammation [19] and its antioxidant properties [21] which actively reduce reactive oxygen species and inhibit cellular damage.

The route of CCl_4_-induced hepatotoxicity is partially dependent on the partial pressure of oxygen in tissues, greatly diminished partial pressure results in predominant formation of trichloromethyl and dichloromethyl radicals and covalent metabolite binding [[22], [39]]. This affects lipid metabolism majorly (increased synthesis, diminished transport out of hepatocytes) and leads to steatosis, or fatty liver. Conversely, raised oxygen partial pressure swings CC1_4_ metabolism towards generation of trichloromethylperoxyl radical with subsequent lipid peroxidation, basically moving the cell from steatosis to apoptosis [22], [23].

Dyslipidemia was caused by CCl_4_ as seen in the results for lipid profile. Silymarin on the other hand reduced LDL to a value that was not significantly different from control value in treated rats. Silymarin reportedly carries this out by up regulating the LDL receptor [24] and stimulating fatty acid β-oxidation, which in turn reduces hepatic triglyceride biosynthesis [25] resulting in reduced LDL and total cholesterol [26]. Triglyceride level was significantly reduced in silymarin treated rats. Increased HDL in silymarin treated groups might be due to silymarin’s polyphenolic constituents which increased liver secretion of apoA-I [27].

Carbon tetrachloride-induced oxidative stress and lipid peroxidation was observed. This is caused by the free radicals trichloromethyl and trichloromethylperoxyl generated from CCl_4_ by cytochrome P 450 enzyme [28]. The lipid peroxidation marker, MDA had its level significantly increased in CCl_4_ group in comparison with control. Malondialdehyde is an end product yielded by the action of free radical on polyunsaturated fatty acids present in biological membranes, and is majorly employed for investigating lipid peroxidation. Its build up in large amounts, as is the case in CCl_4_-injured livers, suggests the failure of endogenous antioxidant systems to halt the generation of more toxic radicals, resulting in gradual peroxidation and consequent hepatic tissue destruction [29]. This also leads to serious alteration of calcium homeostasis and subsequently necrotic cell death [30], [31]. Silymarin reduced MDA in S+CCl_4_ group to a value that is not significantly different from control value. This is majorly ascribed to its antioxidant and free radical scavenging capability. Its antioxidant and cytoprotective property are due to increased glutamine cellular concentrations that stabilize superoxide dismutase and glutathione peroxide. It also inhibits generation of leukotrienes as well as free radicals in Kupffer cells of the liver, thus preventing inflammation and liver enlargement [19]. Glutathione works in concert with antioxidant enzymes to detoxify superoxide anions and hydrogen peroxide in cells [32]. Diminished concentration of GSH plays a major part in the initiation of liver necrosis [33]. Glutathione was significantly reduced in CCl_4_ group while its increment by silymarin in the treated group was not significant.

Oxidative stress elicited by CCl_4_ might be responsible for the significant rise in nitrite seen in CCl_4_ and S+CCl_4_ groups when compared with control group. Nitrite is a reactive oxygen specie that reacts with superoxide anion, forming highly aggressive peroxynitrite radical, which is capable of causing cytotoxicity and DNA damage via lipid peroxidation [34]. Silymarin did not have significant impact on nitrite levels. However, superoxide dismutase and catalase activities were significantly diminished in CCl_4_ group in comparison with control. Silymarin and S+CCl_4_ groups showed significant rise in the activities of both enzymes in comparison with CCl_4_ group and this might be due to silymarin’s ability to accelerate transcription of genes that codes for these enzymes in rat liver [35].

Liver histopathology showed periportal inflammation with infiltrating cells in CCl_4_ group which suggests necrosis. Acute CCl_4_ administration has been reported to produce pericentral liver necrosis, lipid peroxidation and accumulation [36]. Silymarin prevented inflammation in S+CCl_4_ group. This and its restoration of liver enzymes is attributed to its ability to reduce inflammation [19] and its antioxidant property [21] thereby reducing reactive oxygen species and inhibiting cellular damage.

The results also showed an increase in erythrocyte osmotic fragility in CCl_4_ group in comparison with control group indicating that the oxidative stress induced by CCl_4_ may be responsible for instability of red cell membrane and increased haemolysis of red blood cells. This was not significantly reduced by silymarin.

## 5 Conclusion

Findings of this study showed that CCl_4_ induced hepatotoxicity is associated with increased erythrocyte osmotic fragility and disruption of coagulation profile, and pretreatment with silymarin attenuated most of these health derangements in Wistar rats.

## Sources of Funding

This work was funded entirely by the authors.

## CRediT authorship contribution statement

The authors’ responsibilities were as follows – **Aminat B. Popoola, Adesoji A. Fasanmade**: Conceptualization. **Aminat B. Popoola, Emmanuel O. Ademilusi**: Data curation, Formal analysis. **Aminat B. Popoola**: Funding. **Adesoji A. Fasanmade**: Supervision. **Emmanuel O. Ademilusi**: Writing − original draft. **Temitope G. Adedeji**: Writing − review & editing; and all authors: read and approved the final paper.

## Declaration of Competing Interest

The authors declare that they have no known competing financial interests or personal relationships that could have appeared to influence the work reported in this paper.
